# Infective Endocarditis: A Rare Trigger of Immunoglobulin A Vasculitis in an Adult

**DOI:** 10.7759/cureus.9892

**Published:** 2020-08-20

**Authors:** Naga Vaishnavi Gadela, Dimitrios Drekolias, Alain Rizkallah, Jason Jacob

**Affiliations:** 1 Internal Medicine, University of Connecticut, Farmington, USA; 2 Cardiology, Hartford Hospital, Hartford, USA; 3 Internal Medicine, Hartford Hospital, Hartford, USA

**Keywords:** infective endocarditis, iga vasculitis

## Abstract

Immunoglobulin A vasculitis (IgA vasculitis) is a small-vessel vasculitis usually triggered by bacterial or viral infections, antibiotics, and vaccinations. Although it is a disease of the pediatric population, it can occur in adults as well. We present a case of IgA vasculitis that was triggered by underlying infective endocarditis (IE). IE is a rare and fatal cause of the vasculitis that requires timely diagnosis and management to prevent catastrophic outcomes. Our patient was treated with antibiotics for IE, which led to the resolution of vasculitis.

## Introduction

Immunoglobulin A (IgA) vasculitis is characterized by non-thrombocytopenic palpable purpura, arthritis or arthralgias, abdominal pain, and renal disease. A variety of infectious and chemical triggers have been recognized, but the specific underlying pathogenesis remains yet to be elucidated. Current evidence points toward a combination of immunological, genetic, and environmental factors [1]. Although it primarily affects the pediatric population and is often self-limited, around 10% of cases can occur in adults with features and outcomes that may vary. The most common triggers in children include upper respiratory or gastrointestinal infections. We present a case of IgA vasculitis that was triggered by underlying infective endocarditis (IE) in an adult.

## Case presentation

A 55-year-old male with a history of hypertension presented to our hospital with a five-day history of bilateral lower extremity rash that started in his ankles and progressed to involve the upper extremities, trunk, soles, and palms. The patient reported having persistent diarrhea with a 40-pound weight loss over a month. His symptoms were associated with subjective fevers, night sweats, arthralgias, and tea-colored urine. He denied the use of new medications, insect bites, or sick contacts. On presentation, he was afebrile and the exam was notable for a pansystolic grade III/VI murmur over the apex. His upper and lower extremities had a palpable purpuric rash with the involvement of the trunk, gluteal, and intertriginous areas. 

**Figure 1:**
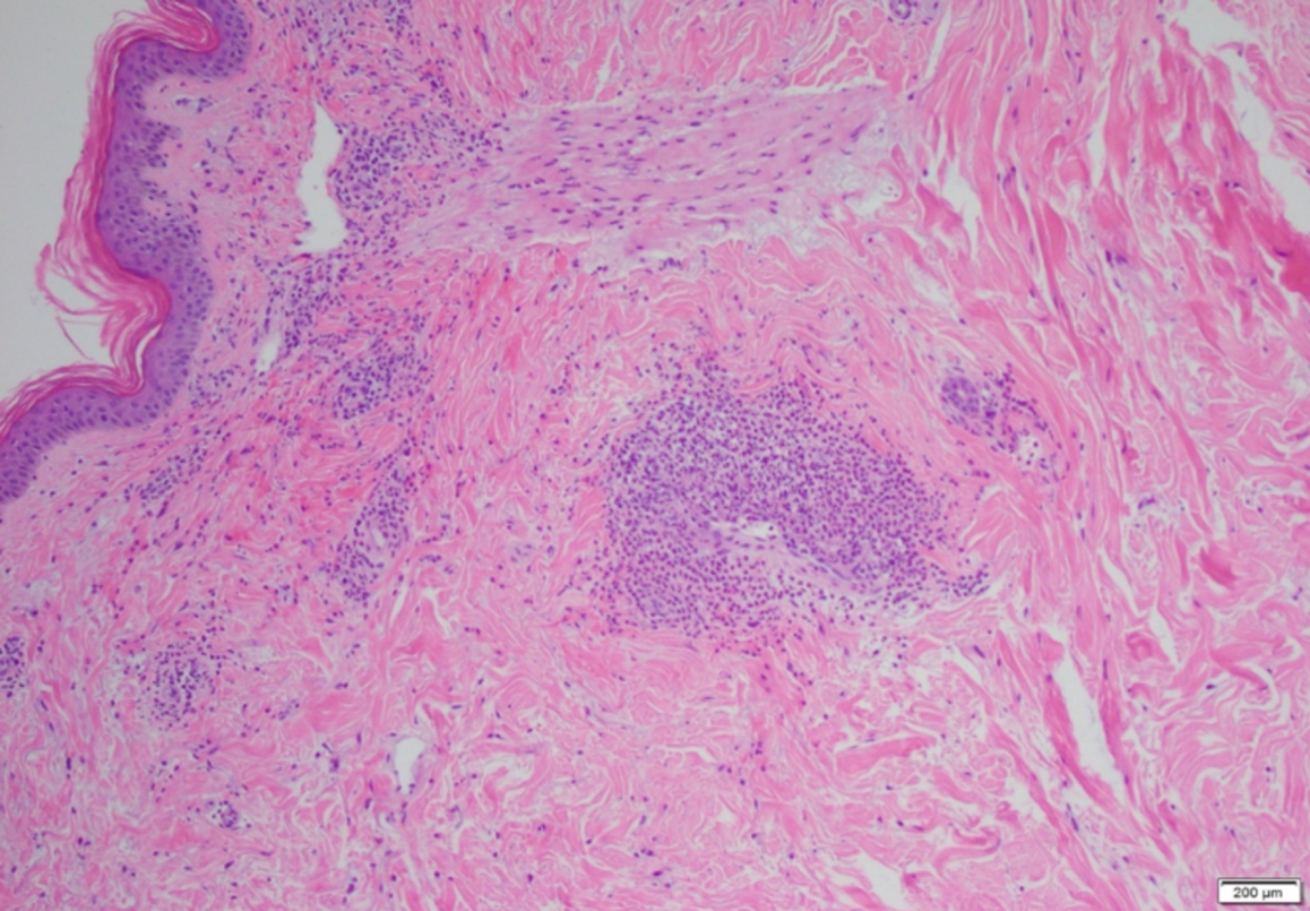
Acute inflammation invading the wall of the vessels.

**Figure 2:**
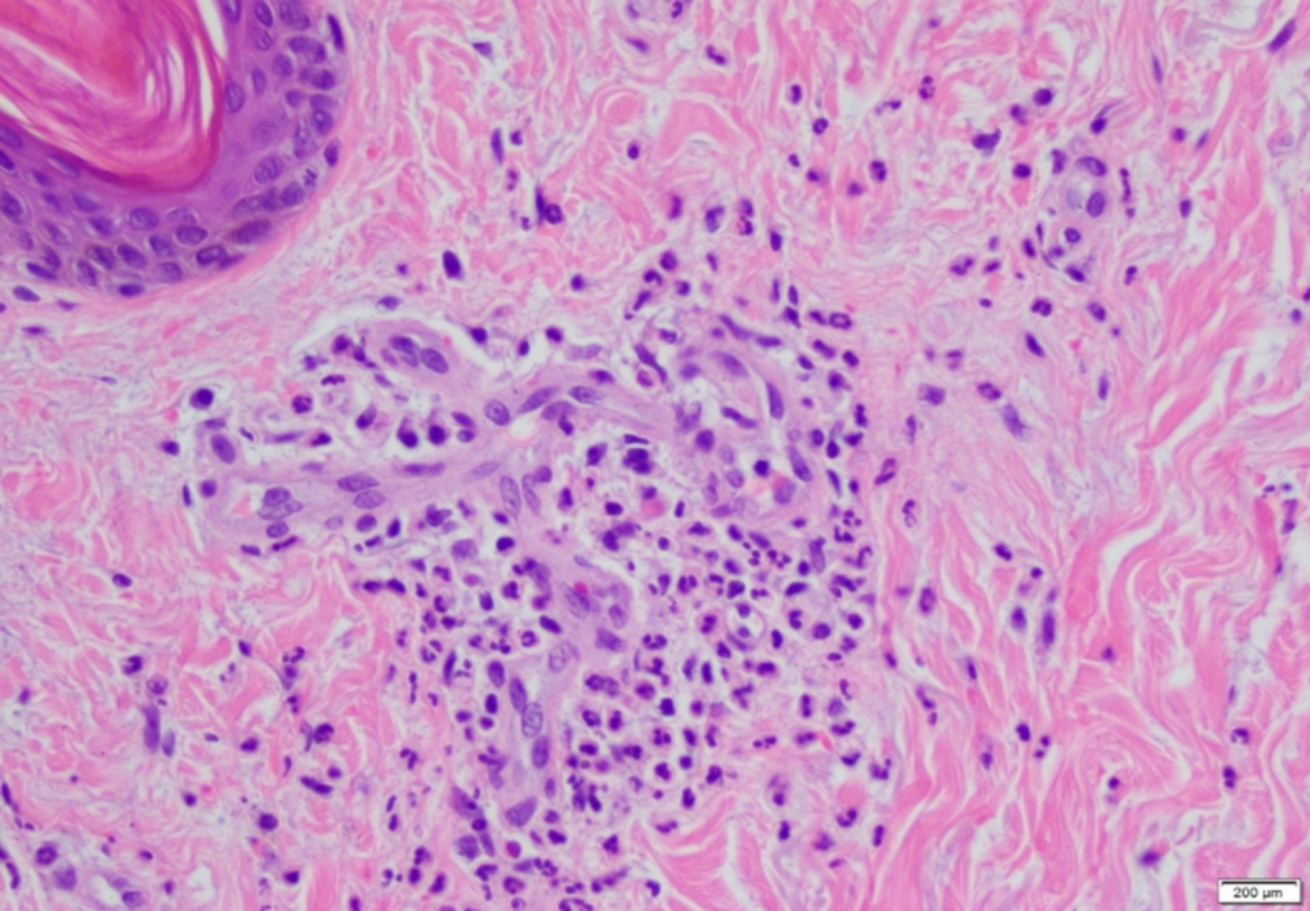
Skin punch biopsy showing leukocytoclastic vasculitis, with neutrophilic infiltrate surrounding and invading the vessels of the superficial vascular plexus. Fibrinoid necrosis of the vessel wall and nuclear debris in association with extravasated erythrocytes is present.

Laboratory investigations were significant for creatinine of 3 mg/dL, urinalysis showing moderate proteinuria of 10 mg/dL, and hematuria > 25 RBCs/high power field with granular casts. Since his presentation was suspicious for IgA vasculitis, rheumatologic testing was performed without any significant results. Infectious workup revealed blood cultures with *Streptococcus viridans*. Transthoracic echocardiography demonstrated large vegetations on the mitral leaflets with severe mitral regurgitation, and the patient was started on antibiotics. Punch biopsy of the purpuric lesions showed evidence of IgA-mediated small-vessel vasculitis (Figures 1, 2). Immunofluorescence studies were consistent with IgA vasculitis with perivascular IgA and immunoglobulin M (IgM) deposition.

The patient was initially started on steroids for the vasculitis; however, it was discontinued due to the presence of an infection. The trigger for IgA vasculitis was attributed to IE, and the patient was managed with antibiotics and supportive therapy, which led to the resolution of vasculitis.

## Discussion

IgA vasculitis primarily affects the pediatric population, but around 10% of cases occur in adults [2]. Bacterial infections activate an immune cascade, leading to the formation of antigen-antibody complexes, which subsequently deposit in the small vessels. These complexes activate the complement pathway, which further leads to neutrophilic activation resulting in vasculitis [3]. The most common triggers include mucosal infections; however, IE as a trigger for IgA vasculitis is very rare, with less than 10 cases reported in the literature [4]. In our patient, the vasculitis resolved after the treatment of endocarditis, which further supports the association. In a study by Loricera et al., among the 766 patients who presented with cutaneous vasculitis, 6 patients were diagnosed with an underlying IE, which triggered the vasculitis [5]; hence, it is important to assess for the presence of an underlying bacterial infection as the management could vary significantly. Although skin lesions often occur with endocarditis, it is vital to differentiate them from the skin lesions of vasculitis. In IE, skin lesions usually manifest as hemorrhagic/petechial lesions rather than palpable purpura [6]. Immunofluorescence is the primary modality for diagnosing and may demonstrate IgA, complement component 3, and fibrin deposition within the walls of the vessels. The characteristic histological finding of IgA vasculitis is leukocytoclastic vasculitis, with IgA complexes deposited within the vessels.

There are minimal data in the form of randomized controlled trials to guide the management of IgA vasculitis in adults. The treatment is usually supportive unless there is nephritis, in which case the management depends on the severity of renal involvement. In adults with limited renal involvement and if proteinuria is present, angiotensin-converting enzyme (ACE) inhibitors/angiotensin II receptor blockers (ARBs) are used to reduce proteinuria, unless otherwise contraindicated. If more severe renal manifestations are present with marked proteinuria of >1 g/day, nephrotic syndrome, or crescentic glomerulonephritis, a prolonged course of steroids with tapering (six months) may be indicated to ameliorate the inflammation, although the underlying pathophysiology does not appear to be affected. Our patient was initially placed on steroids, but they were held off in the setting of the active infection. Cyclophosphamide or mycophenolate-mofetil could also be added if there is a significant presence of crescents on kidney biopsy (in the range of 20% to 25%), although it is notable that high-quality evidence for this recommendation is lacking. Observational studies and case series have investigated the use of rituximab in adults with steroid-resistant IgA vasculitis [7-10]. The option of renal transplantation can also be explored for patients who progressed to end-stage renal disease; however, the possibility of recurrent IgA vasculitis cannot be excluded. Of note, in most cases, the IgA deposition that occurs in the new graft does not progress to overt clinically apparent IgA vasculitis [11-15].

## Conclusions

Although fever is one of the most prominent features of IE, it should be noted that our patient did not have a fever. Hence, physicians must be mindful of not excluding the possibility of an underlying IE because of the absence of fever. Our case highlights the importance of physical examination. The initial evidence supporting the diagnosis of IE was elicited by the presence of murmur, which led to further investigation. It would be beneficial to have a broad differential and a high suspicion for IE when an adult presents with purpuric lesions and renal failure, and work-up for coagulation disorders is not yielding a diagnosis.

## References

[1] Roache-Robinson P, Hotwagner DT (2020). Henoch Schonlein Purpura (Anaphylactoid Purpura, HSP). https://europepmc.org/article/NBK/NBK537252.

[2] Kopparapu A, Jarrett D, Kraleti S (2019). IgA vasculitis presenting as abdominal pain and rash. Proc (Bayl Univ Med Cent).

[3] Sohagia AB, Gunturu SG, Tong TR (2010). Henoch-schonlein purpura-a case report and review of the literature. Gastroenterol Res Pract.

[4] Wang JX, Perkins S, Totonchy M (2020). Endocarditis-associated IgA vasculitis: two subtle presentations of endocarditis caused by Candida parapsilosis and Cardiobacterium hominis. JAAD Case Rep.

[5] Loricera J, Blanco R, Hernandez JL (2015). Cutaneous vasculitis associated with severe bacterial infections. A study of 27 patients from a series of 766 cutaneous vasculitis. Clin Exp Rheumatol.

[6] Galaria NA, Lopressti NP, Magro CM (2002). Henoch-Schonlein purpura secondary to subacute bacterial endocarditis. Cutis.

[7] Maritati F, Fenoglio R, Pillebout E (2018). Brief report: rituximab for the treatment of adult-onset IgA vasculitis (Henoch-Schonlein). Arthritis Rheumatol.

[8] Donnithorne KJ, Atkinson TP, Hinze CH (2009). Rituximab therapy for severe refractory chronic Henoch-Schonlein purpura. J Pediatr.

[9] Fenoglio R, Naretto C, Basolo B (2017). Rituximab therapy for IgA-vasculitis with nephritis: a case series and review of the literature. Immunol Res.

[10] Ishiguro H, Hashimoto T, Akata M (2013). Rituximab treatment for adult purpura nephritis with nephrotic syndrome. Intern Med.

[11] Ponticelli C, Moroni G, Glassock RJ (2011). Recurrence of secondary glomerular disease after renal transplantation. Clin J Am Soc Nephrol.

[12] Kanaan N, Mourad G, Thervet E (2011). Recurrence and graft loss after kidney transplantation for henoch-schonlein purpura nephritis: a multicenter analysis. Clin J Am Soc Nephrol.

[13] Hasegawa A, Kawamura T, Ito H (1989). Fate of renal grafts with recurrent Henoch-Schonlein purpura nephritis in children. Transplant Proc.

[14] Nast CC, Ward HJ, Koyle MA, Cohen AH (1987). Recurrent Henoch-Schonlein purpura following renal transplantation. Am J Kidney Dis.

[15] Meulders Q, Pirson Y, Cosyns JP, Squifflet JP, van Ypersele de Strihou C (1994). Course of Henoch-Schonlein nephritis after renal transplantation. Report on ten patients and review of the literature. Transplantation.

